# Development of a nomogram model for predicting coronary heart disease in patients with metabolic-associated fatty liver disease

**DOI:** 10.3389/fcvm.2025.1652321

**Published:** 2025-09-23

**Authors:** Zhengliang Li, Xiaokai Chen, Juan Wang, Weirui Chen, Run Zhang, Lihua Cao, Shaoting Shi, Linlin Ren, Wenzhong Zhang

**Affiliations:** ^1^ Department of Cardiology, The Affiliated Hospital of Qingdao University, Qingdao, China; ^2^ Department of Gastroenterology, The Affiliated Hospital of Qingdao University, Qingdao, China

**Keywords:** metabolic-associated fatty liver disease, coronary atherosclerotic heart disease, triglyceride-glucose index, insulin resistance, nomogram

## Abstract

**Objective:**

To investigate the risk factors associated with coronary heart disease (CHD) in patients with metabolic-associated fatty liver disease (MAFLD) and develop a nomogram prediction model.

**Methods:**

This study included 394 patients with MAFLD who underwent coronary angiography at The Affiliated Hospital of Qingdao University between December 2019 and December 2024. The study cohort was divided in a 7:3 ratio into training and validation sets comprising 277 and 117 cases, respectively. The training group was further divided into the MAFLD-only (*n* = 57) and MAFLD-plus-CHD (*n* = 220) groups. LASSO and multivariable logistic regression analyses were performed to identify the risk factors of concomitant coronary heart disease in patients with MAFLD. A nomogram was constructed and validated internally to predict CHD risk in the patients. We evaluated the nomogram's predictive performance using receiver operating characteristic (ROC) curves, calibration plots, and decision curve analysis (DCA) in the training and validation groups.

**Results:**

Of the 394 MAFLD cases, 313 had CHD-related complications. Of the 277 patients in the training set, 220 had CHD, and of the 117 patients in the validation set, 93 had CHD. LASSO regression analysis revealed that the following variables were associated with the risk of CHD: sex, lipoprotein(a) (Lp[a]), low-density lipoprotein cholesterol, white blood cell count (WBC), glycated triglyceride-glucose index (TyG), and atherosclerosis index (AIP). Multivariate logistic regression analysis revealed that sex, Lp(a), WBC, TyG, and AIP were independent risk factors for CHD in MAFLD cases. A nomogram was constructed and an ROC curve was plotted, based on which the optimal cutoff value was determined as 0.698. The area under the curve of the nomogram in the training and validation cohorts was 0.860 (95% CI = 0.807–0.913) and 0.843 (95% CI = 0.757–0.929), respectively. Calibration curves for CHD risk probability showed good agreement between the nomogram's predicted probabilities and the observed event rates. DCA demonstrated the net clinical benefit of the constructed nomogram.

**Conclusion:**

Sex, Lp(a), WBC, TyG, and AIP emerged as independent risk factors for CHD in patients with MAFLD and the nomogram prediction model constructed using these factors could effectively predict CHD occurrence.

## 1 Introduction

Metabolic-associated fatty liver disease (MAFLD) is a chronic metabolic stress-related liver disease that occurs in genetically predisposed individuals and is associated with nutritional overload and insulin resistance (IR) (1). In developed countries, such as those in Europe and North America, the prevalence of MAFLD continues to rise. In China, too, MAFLD has shown a steady increased in recent years and is projected to surpass viral hepatitis as the leading chronic liver disease (1–3).

Coronary heart disease (CHD), the most prevalent cardiovascular disease (CVD), is the leading cause of chronic disease-related mortality globally (4). Atherosclerosis and the development of arterial plaques are central to the pathophysiology of CHD (5). Additionally, inflammatory responses, oxidative stress, disruptions in glucose and lipid metabolism, and endothelial dysfunction contribute significantly to the development and progression of CHD (6, 7). A large number of recent studies indicate that IR plays a crucial role in coronary plaque formation and remodeling, independent of traditional risk factors such as age, smoking, genetic susceptibility, obesity, and hypertension (HTN) (8, 9).

Studies have shown that the presence and severity of MAFLD are associated with increased risk of CVD (10), and CVD is the leading cause of death in patients with MAFLD (11).

Based on recent research into the pathogenesis of MAFLD and CHD, it has become evident that both diseases share multiple risk factors (12, 13). Beyond traditional factors, IR and dyslipidemia play vital roles in patients with MAFLD (14). The triglyceride-glucose (TyG) index has emerged as a reliable surrogate marker for IR diagnosis (15). Recent research has identified the TyG index as a novel independent predictor of CHD and a strong indicator of other cardiovascular outcomes (16). In addition, there are other important mechanisms that contribute to atherosclerosis and could be potential risk markers (17, 18). For example, atherogenic index of plasma (AIP), which combines serum triglyceride (TG) and high-density lipoprotein cholesterol (HDL-C), more accurately precisely reflects the pathogenicity and specificity of dyslipidemia (19) and has been positively associated with MAFLD risk, making it a potential predictive marker for MAFLD (20). There is currently no evidence that clearly indicates the relationship between AIP and CHD in the context of MAFLD. In addition to AIP, lipoprotein(a) (Lp[a]), a routine lipid-related biomarker, has recently been identified as a key predictor of increased cardiovascular risk in healthy American women over 30 year of age (21).

In China, MAFLD presents several challenges, including an increasing number of cases, a strong genetic predisposition, a growing incidence in younger populations, and a lack of simple diagnostic and treatment tools. This study aims to identify accessible and accurate laboratory indicators and construct an effective, practical prediction model to facilitate early clinical identification and intervention for patients with MAFLD who are at risk of developing CHD in the Chinese population, in order to improve their quality of life.

## 2 Materials and methods

### 2.1 Study population

A total of 456 patients with MAFLD who underwent coronary angiography at the Affiliated Hospital of Qingdao University between December 2019 and December 2024 were screened for eligibility. The inclusion criteria were as follows: (1) diagnosis of hepatic steatosis by certified sonographers using standard methods (22) and (2) presence of ≥1 cardiovascular risk factor (obesity, hypertension, diabetes, hypertriglyceridemia, or low HDL-C levels) in addition to hepatic steatosis. The exclusion criteria were: (1) history of excessive alcohol consumption (men >140 g/week, women >70 g/week), viral hepatitis, or use of hepatotoxic drugs; (2) prior use of antiplatelet or lipid-lowering therapy; (3) history of percutaneous coronary intervention or coronary artery bypass grafting; (4) presence of other cardiac conditions; (5) malignancy, autoimmune disorders, acute or chronic infections, or severe cerebrovascular disease. CHD was diagnosed according to Judkins method and was considered present if ≥50% luminal stenosis was detected in any major coronary artery or a significant branch on as visualized by coronary angiography (23).

**Figure 1:**
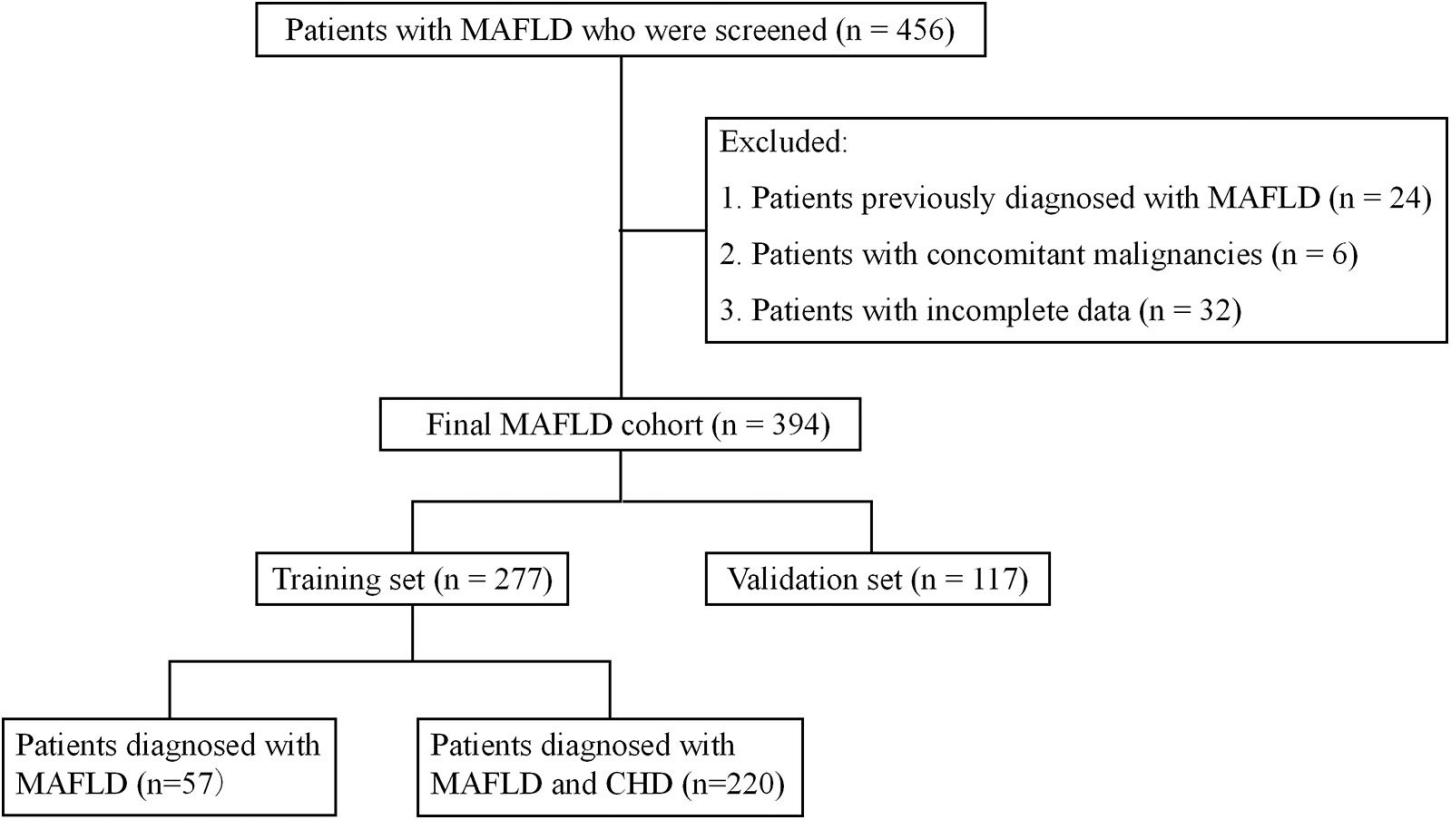
Schematic depicting patient selection and grouping.

Based on the above criteria, 394 eligible patients were included and randomly assigned to a training set and a validation set at a 7:3 ratio (277 in the training set and 117 in the validation set). The training set was subdivided into the MAFLD-only group (*n* = 57) and the MAFLD + CHD group (*n* = 220) based on the results of coronary angiography (Figure 1).

This study was approved by the Ethics Committee of the Affiliated Hospital of Qingdao University, and written informed consent was obtained from all participants.

### 2.2 Data collection and calculation of indices

Basic demographic and laboratory data of the included patients were retrieved from the hospital's electronic medical records. The collected variables included age, sex, height, weight, CHD status, history of hypertension, diabetes, smoking, and alcohol use. The laboratory parameters included serum albumin (Alb), fasting blood glucose (FBG), glycated hemoglobin (HbA1c), alanine aminotransferase (ALT), aspartate aminotransferase (AST), TG, total cholesterol (TC), Lp(a), HDL-C, low-density lipoprotein cholesterol (LDL-C), free fatty acids (FFA), blood urea nitrogen (BUN), serum creatinine (Scr), cystatin C (Cys-C), uric acid (UA), complete blood count, and other indices.

The following formulas were used to calculate some of the indices:

$$BMI=\mathrm{weight}(\mathrm{kg})/[\mathrm{height}(m){]}^{2}$$

NLR(neutrophil-to-lymphocyteration)=neutrophilcount/lymphocytecount

SII(systemicimmune-inflammationindex)=plateletcount×neutrophilcount/lymphocytecount

PNI(prognosticnutritionalindex)=Alb(g/L)+5×lymphocytecount

TyGindex=ln[(FBG×TG)/2]

HSI(hepaticsteatosisindex)=(AST/ALT×plateletcount)/Alb

AIP=ln(TG/HDL-C)

### 2.3 Statistical analysis

Statistical analyses were performed using R (v4.3.3) and SPSS (v27.0.0). The createDataPartition function in R was used for random sampling. The normality of continuous variables was tested using the Shapiro–Wilk test. Variables following a normal distribution were expressed as mean ± SD and compared using the *t*-test; non-normally distributed variables were expressed as median (interquartile range) and compared using the Mann–Whitney *U* test. Categorical variables were compared using the chi-square test.

LASSO regression was performed in the training cohort to identify risk factors for CHD in patients with MAFLD. Statistically significant variables were included in multivariate logistic regression to identify independent predictors, which were used to construct a nomogram with the nomogram function in R. Receiver operating characteristic (ROC) curves were plotted, and area under the curve (AUC) was calculated to assess model discrimination. Calibration was evaluated using calibration curves and the Hosmer–Lemeshow test. Clinical utility was assessed using decision curve analysis (DCA). *p*-value <0.05 were considered to indicate statistical significance.

## 3 Results

### 3.1 Baseline characteristics of the training and validation sets

There were no statistically significant differences between the training set (*n* = 277) and validation set (*n* = 117) in terms of sex, age, history of hypertension or diabetes, smoking status, BMI, Alb, FBG, HbA1c, ALT, AST, TG, TC, Lp(a), HDL-C, LDL-C, FFA, BUN, Scr, CysC, UA, WBC, neutrophils count, lymphocytes count, PLT, NLR, SII, PNI, TyG index, HSI, or AIP (*P* > 0.05), as shown in Supplementary Table S1.

In the training set, the proportion of male patients was significantly higher in the MAFLD + CHD (*n* = 220) than in the MAFLD-only group (*n* = 57) (*P* < 0.05). No significant differences were found between the two groups with regard to other baseline characteristics such as hypertension, diabetes, and smoking history (*P* > 0.05). The values of the biochemical indicators including HbA1c, Scr, CysC, WBC, N, L, TyG index, and AIP were all significantly higher in the MAFLD + CHD group than in the MAFLD-only group (*P* < 0.05). No significant differences were observed in the other variables (*P* > 0.05), as detailed in Supplementary Table S2.

### 3.2 LASSO regression analysis of CHD risk factors

**Figure 2:**
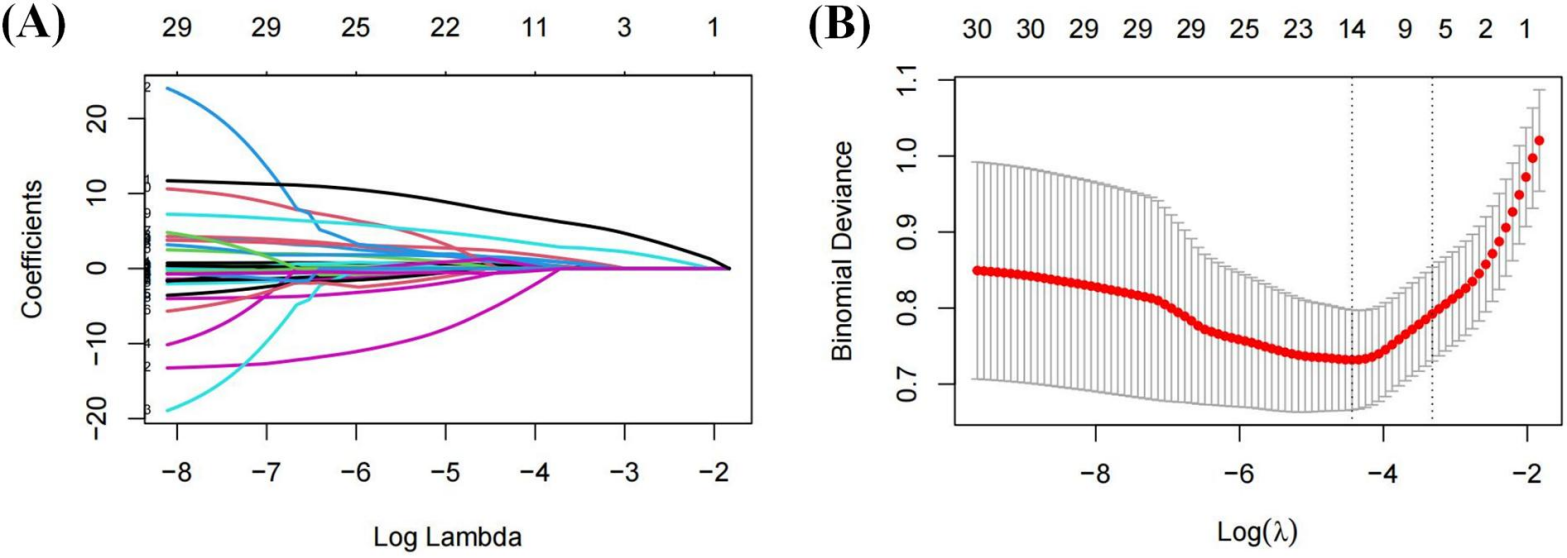
Variable filtering process of LASSO regression: **(A)** coefficient shrinkage path plot; **(B)** cross-validation plot.

A total of 31 variables were analyzed in the training cohort. The variable were coded as 1 or 0, as shown in these examples: MAFLD + CHD = 1, MAFLD = 0; male = 1, female = 0. LASSO regression analysis was performed to select significant predictors of CHD. The coefficient paths and cross-validation curves are shown in Figure 2. Using 5-fold cross-validation, the optimal penalty parameter (*λ*) was determined. At a *λ*.min value of = 0.012, 14 variables were retained. However, to achieve a more parsimonious model, *λ*.1se value of = 0.036 was selected, based on which 6 predictors were retained with minimal loss in model accuracy.

The final selected predictors included sex, Lp(a), LDL-C, WBC, TyG index, and AIP.

### 3.3 Multivariate logistic regression analysis of CHD risk factors

The six variables identified from LASSO regression were entered into a multivariate logistic regression model. The results showed that sex, Lp(a), WBC, TyG index, and AIP were independent risk factors for CHD in patients with MAFLD. Detailed statistics are presented in Supplementary Table S3.

### 3.4 Construction and validation of the CHD prediction nomogram

**Figure 3:**
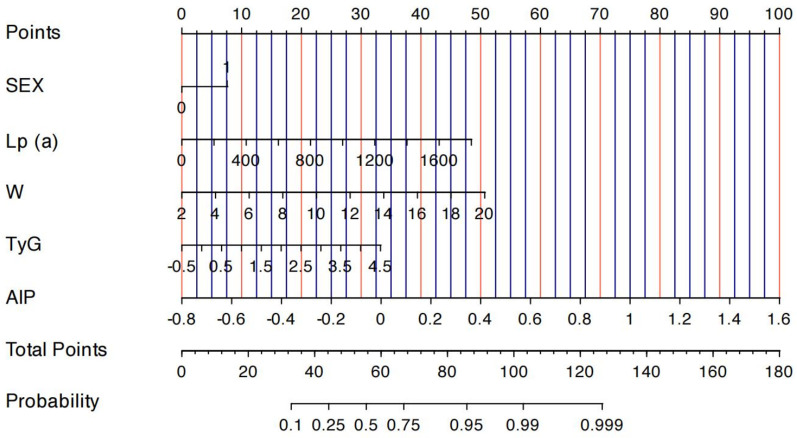
Nomogram depicting the risk of developing CHD in patients with MAFLD. To use the nomogram, draw an upward vertical line from each covariate to the points bar to calculate the number of points. Based on the sum of the covariate points, draw a downward vertical line from the total points line to calculate the probability of developing CHD.

Based on the results of the multivariate logistic regression, a nomogram incorporating the five identified independent risk factors—sex, Lp(a), WBC, TyG index, and AIP—was developed to predict CHD risk in MAFLD patients (Figure 3). Each variable was assigned a point score, and the total score was used to estimate the probability of developing CHD. A total score above 60 was considered to indicated CHD risk >50%, and a score above 88, CHD risk >95%.

### 3.5 Internal validation and model performance

**Figure 4:**
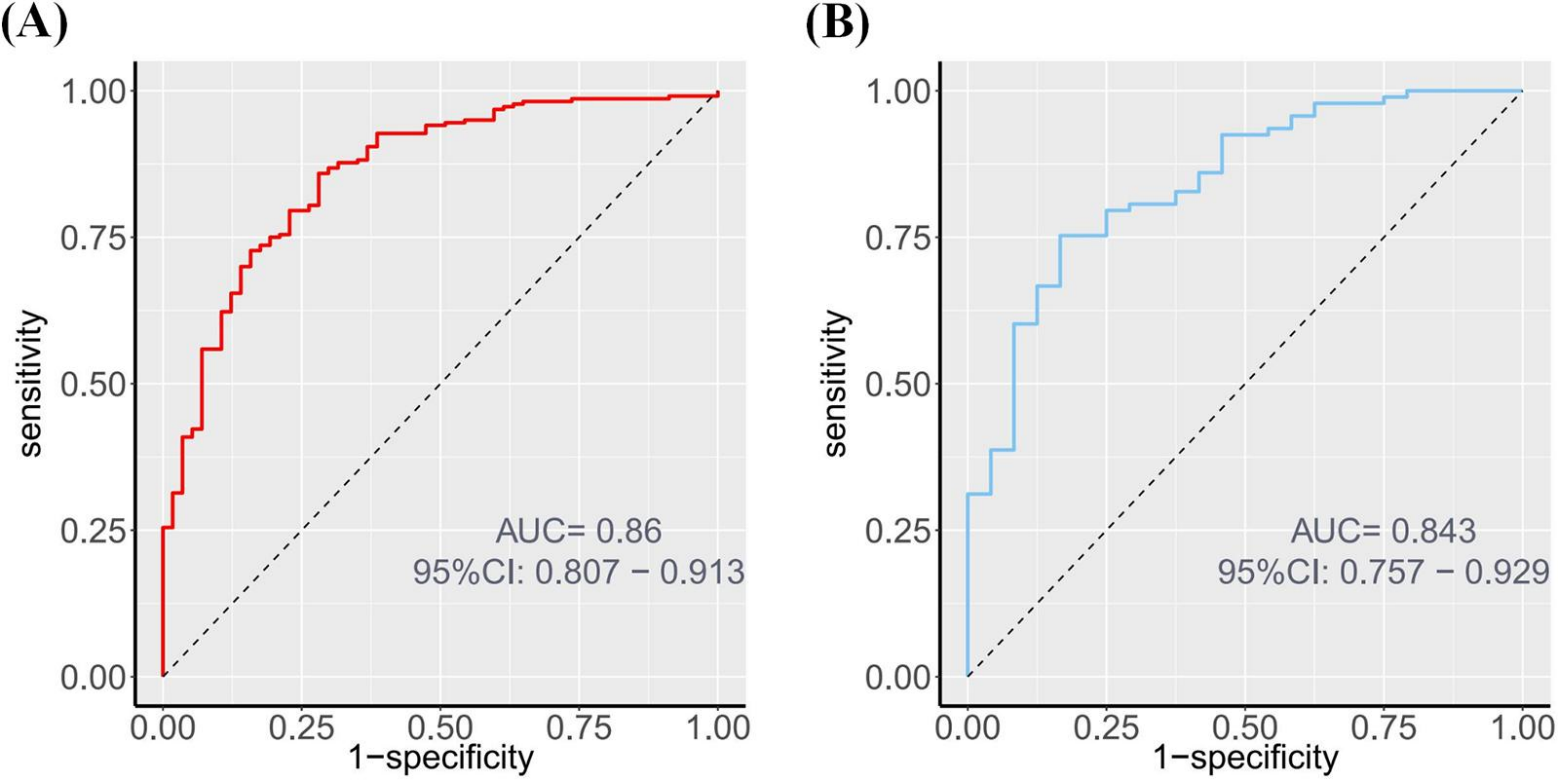
ROC of the nomogram. **(A)** ROC curve depicting the discrimination ability of the nomogram in the training set, with an AUC of 0.860. **(B)** ROC curve depicting the discrimination ability of the nomogram in the validation set, with an AUC of 0.843.

The nomogram's performance was evaluated using ROC curves in both the training and validation sets. The AUC was 0.860 (95% CI: 0.807–0.913) in the training set and 0.843 (95% CI: 0.757–0.929) in the validation set (Figures 4A,B), These AUC values indicate excellent discriminatory ability.

**Figure 5:**
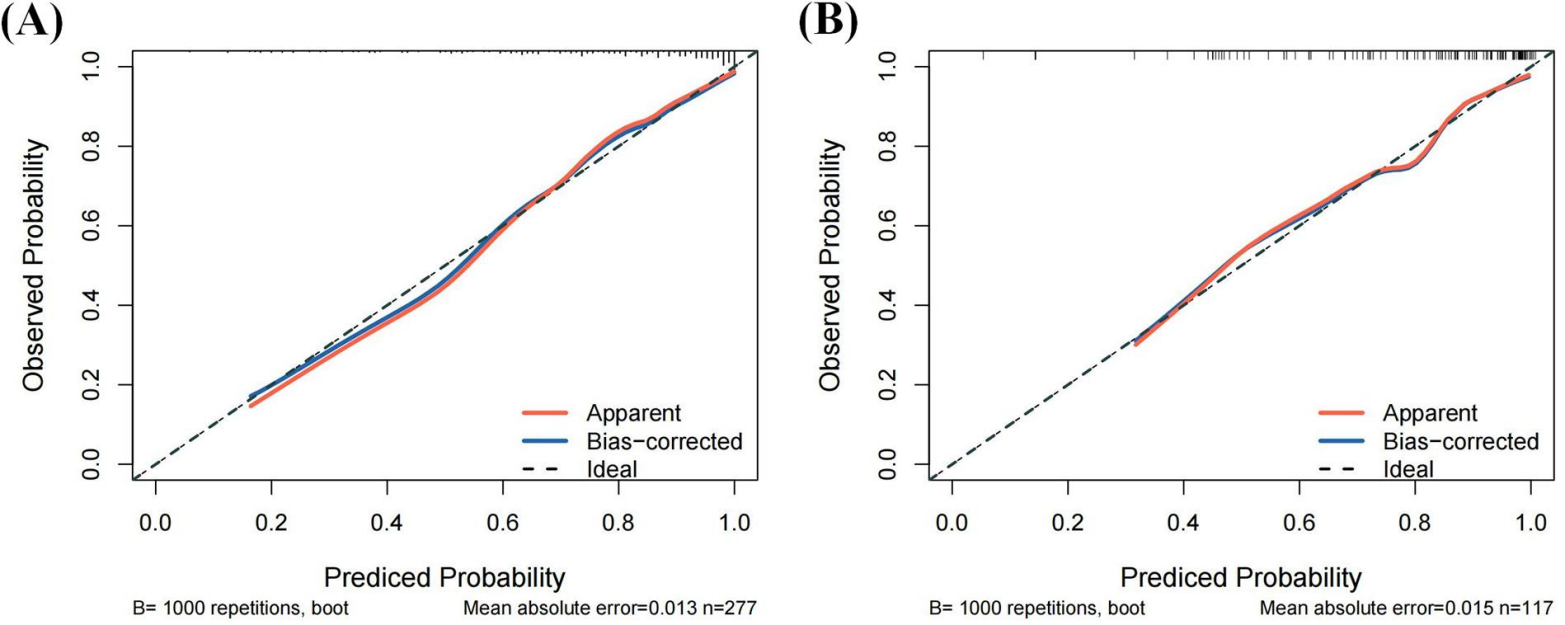
Calibration curves of the nomogram for predicting CHD from the training cohort and the validation cohort. **(A)** Calibration curves of the nomogram for predicting CHD from the training cohort. The Hosmer–Lemeshow test had a *P*-value of 0.122 in the training cohort; **(B)** Calibration curves of the nomogram for predicting CHD from the validation cohort. The Hosmer–Lemeshow test had a *P*-value of 0.465 in the validation cohort.

Calibration plots showed good agreement between the probabilities and observed event rates (Figure 5). The Hosmer–Lemeshow test indicated good model fit in both the training (*P* = 0.808) and validation (*P* = 0.630) sets.

**Figure 6:**
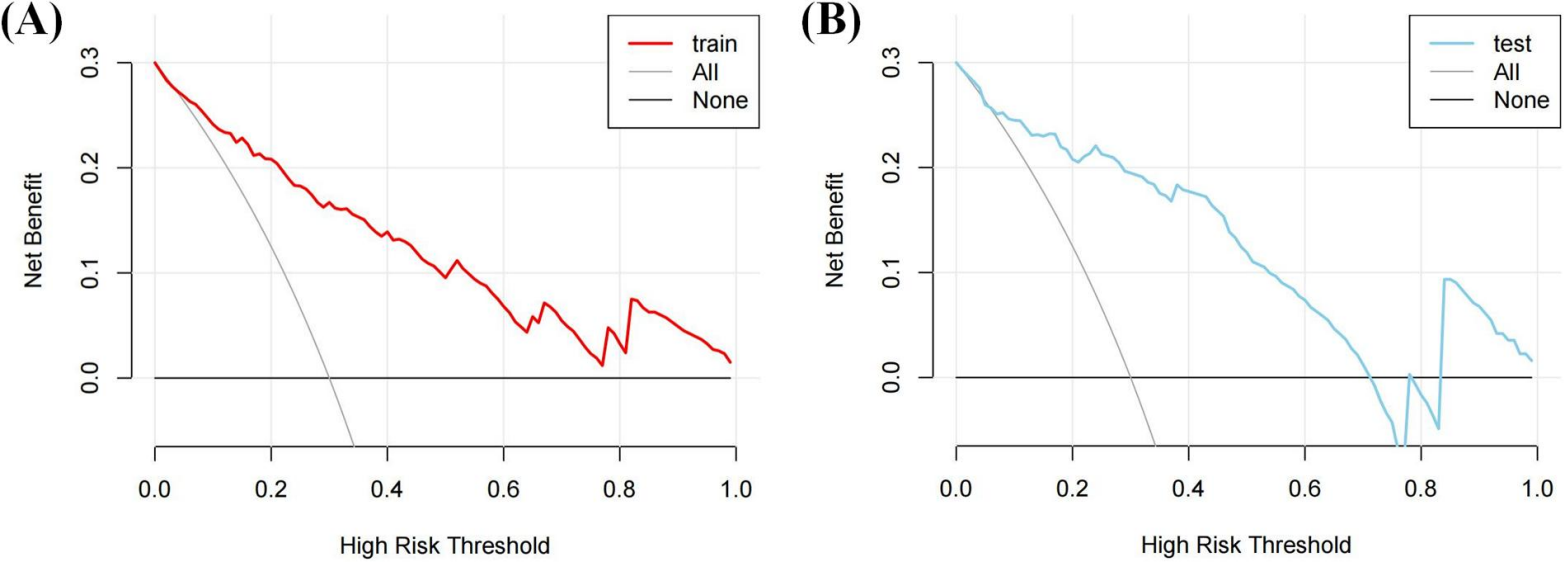
DCA of the nomogram. **(A)** The DCA curve of the training cohort; **(B)** The DCA curve of the validation cohort.

DCA (Figure 6) demonstrated that the nomogram provided a higher net clinical benefit than either treating all patients or none when the threshold probability exceeded 30%, indicating good clinical utility.

## 4 Discussion

This study developed and validated a practical nomogram based on routine laboratory parameters to predict the risk of CHD in patients with MAFLD. The model achieved high predictive accuracy with AUCs of 0.860 and 0.843 in the training and validation sets, respectively. Given that MAFLD patients have approximately 50% higher cardiovascular mortality than the general population (24), and given the often silent clinical course of the disease, this model provides a valuable tool for early identification of high-risk patients. Importantly, all the incorporated predictors—sex, Lp(a), WBC, TyG index, and AIP—are routinely assessed in clinical practice, making the model highly feasible for real-world application.

From a mechanistic standpoint, this study highlights multiple intersecting pathways linking MAFLD and CHD. **Metabolic imbalance:** The associations of the TyG index and AIP with CHD risk support the pivotal role of IR in disease development, consistent with previous cohort studies (9, 25–27). **Inflammation:** Elevated WBC levels reflect systemic low-grade inflammation, supporting the use of WBC count in cardiovascular risk stratification in MAFLD, in alignment with the conceptual framework proposed by Jiang et al. (28). **Lipoprotein(a):** Its inclusion enhances risk assessment for atherosclerosis and emphasizes the need for personalized monitoring given its >90% genetic determination (29).

### 4.1 Strengths of the study

The new MAFLD diagnostic criteria used to construct the count risk prediction model is in better alignment with the metabolic origins of the disease. Accordingly, the integration of hepatic, cardiovascular, and metabolic markers, yielded higher performance (AUC = 0.860) than traditional markers such as the Fibrosis-4 index (30). Moreover, the model demonstrated strong generalizability with an AUC of 0.843 in the validation cohort, thus outperforming ultrasound-based models (AUC = 0.68–0.80) (31).

### 4.2 Limitations

The lack of histopathological grading limited precision in MAFLD subtype stratification. Further the retrospective nature of the study may have introduced residual confounding. An additional limitation is the use of a single-center dataset, which may restrict the generalizability and external application of the mode. Based on these limitations, future work should include multi-center prospective cohorts and integrate of omics data (e.g., microRNA, and proteomics data) to refine risk stratification. Lp(a) may not be a routine testing parameter in some clinical contexts. We also note that the predictive performance of models excluding Lp(a) requires further validation, and efforts should be made to identify more conventional indicators that could serve as alternatives.

### 4.3 Clinical Implications

This model supports comprehensive MAFLD management in three areas: **primary prevention** through of early identification of high-risk individuals; **therapeutic monitoring** facilitated by dynamic assessment of biomarkers to guide treatment, and **cost-effectiveness** as a result of the utilization of routine markers that lowers barriers to implementation in primary care settings. Finally, with novel Lp(a)-targeted agents, such as pelacarsen, entering phase III trials, this model may aid in precision treatment stratification.

### 4.4 Conclusion

This nomogram, integrates indices related to metabolic dysregulation, inflammation, and lipid abnormalities, to provide a reliable tool for stratifying CHD risk in MAFLD patients. Tn addition it enhances our understanding of MAFLD–CVD comorbidity and offers a foundation for early intervention strategies to prevent cardiovascular events.

## Data Availability

The raw data supporting the conclusions of this article will be made available by the authors, without undue reservation.

## References

[1] Chris E Anstee QM Teresa ALM Heike B Stefeno B Joan C Modeling NAFLD Disease Burden in China, France, Germany, Italy, Japan, Spain, United Kingdom, and United States for the period 2016–2030. J Hepatol. (2018) 69:896–904. 10.1016/j.jhep.2018.05.036 29886156

[2] Le MH Yeo YH Li X Li J Zou B Wu Y 2019 global NAFLD prevalence: a systematic review and meta-analysis. Clin Gastroenterol Hepatol. (2022) 20(12):2809–17.e28. 10.1016/j.cgh.2021.12.002 34890795

[3] Li J Zou B Yeo YH Feng Y Xie X Lee DH Prevalence, incidence, and outcome of non-alcoholic fatty liver disease in Asia, 1999–2019: a systematic review and meta-analysis. Lancet Gastroenterol Hepatol. (2019) 4(5):389–98. 10.1016/S2468-1253(19)30039-1 30902670

[4] Woodruff RC Tong X Khan SS Shah NS Jackson SL Loustalot F Trends in cardiovascular disease mortality rates and excess deaths, 2010–2022. Am J Prev Med. (2024) 66(4):582–9. 10.1016/j.amepre.2023.11.009 37972797 PMC10957309

[5] Shaya GE Leucker TM Jones SR Martin SS Toth PP. Coronary heart disease risk: low-density lipoprotein and beyond. Trends Cardiovasc Med. (2021) 32(4):181–94. 10.1016/j.tcm.2021.04.002 33872757

[6] Cao Y Li P Zhang Y Qiu M Li J Ma S Association of systemic immune inflammatory index with all-cause and cause-specific mortality in hypertensive individuals: results from NHANES. Front Immunol. (2023) 14:1087345. 10.3389/fimmu.2023.1087345 36817427 PMC9932782

[7] Li Z Liu Q Yao Z. The serum uric acid-to-high-density lipoprotein cholesterol ratio is a predictor for all-cause and cardiovascular disease mortality: a cross-sectional study. Front Endocrinol (Lausanne). (2024) 15:1417485. 10.3389/fendo.2024.1417485 39345882 PMC11427315

[8] Libby P. The changing landscape of atherosclerosis. Nature. (2021) 592(7855):524–33. 10.1038/s41586-021-03392-8 33883728

[9] Hill MC Kadow ZA Long H Morikawa Y Martin TJ Birks EJ Integrated multi-omic characterization of congenital heart disease. Nature. (2022) 608:181–91. 10.1038/s41586-022-04989-3 35732239 PMC10405779

[10] Lee HH Lee HA Kim EJ Kim HY Kim HC Ahn SH Metabolic dysfunction-associated steatotic liver disease and risk of cardiovascular disease. Gut. (2024) 73(3):533–40. 10.1136/gutjnl-2023-331003 37907259

[11] Rao SV O'Donoghue ML Ruel M Rab T Tamis-Holland JE Alexander JH 2025 ACC/AHA/ACEP/NAEMSP/SCAI guideline for the management of patients with acute coronary syndromes: a report of the American College of Cardiology/American Heart Association Joint Committee on Clinical Practice Guidelines. Circulation. (2025) 151(13):e771–862. 10.1161/CIR.0000000000001328 40014670

[12] Lee H Lee YH Kim SU Kim HC. Metabolic dysfunction-associated fatty liver disease and incident cardiovascular disease risk: a nationwide cohort study. Clin Gastroenterol Hepatol. (2021) 19(10):2138–47.e10. 10.1016/j.cgh.2020.12.022 33348045

[13] Caussy C Aubin A Loomba R. The relationship between type 2 diabetes, NAFLD, and cardiovascular risk. Curr Diab Rep. (2021) 21(5):15. 10.1007/s11892-021-01383-7 33742318 PMC8805985

[14] Toh JZK Pan XH Tay PWL Ng CH Yong JN Xiao J A meta-analysis on the global prevalence, risk factors and screening of coronary heart disease in nonalcoholic fatty liver disease. Clin Gastroenterol Hepatol. (2022) 20(11):2462–73.e10. 10.1016/j.cgh.2021.09.021 34560278

[15] Vasques ACJ Novaes FS Oliveira MDSD Souza JRM Yamanaka A Pareja JC Tyg index performs better than HOMA in a Brazilian population: a hyperglycemic clamp validated study. Diabetes Res Clin Pract. (2011) 93(3):e98–100. 10.1016/j.diabres.2011.05.030 21665314

[16] Park B Lee YJ Lee HS Jung DH. The triglyceride-glucose index predicts ischemic heart disease risk in Koreans: a prospective study using national health insurance service data. Cardiovasc Diabetol. (2020) 19(1):210. 10.1186/s12933-020-01186-2 33302952 PMC7731566

[17] Cai J Zhang XJ Ji YX Zhang P She ZG Li H. Nonalcoholic fatty liver disease pandemic fuels the upsurge in cardiovascular diseases. Circ Res. (2020) 26(5):679–704. 10.1161/CIRCRESAHA.119.316337 32105577

[18] Huang DQ El-Serag HB Loomba R. Global epidemiology of NAFLD-related HCC: trends, predictions, risk factors and prevention. Nat Rev Gastroenterol Hepatol. (2020) 18(4):223–38. 10.1038/s41575-020-00381-6 33349658 PMC8016738

[19] Yin B Wu Z Xia Y Xiao S Chen L Li Y. Non-linear association of atherogenic index of plasma with insulin resistance and type 2 diabetes: a cross-sectional study. Cardiovasc Diabetol. (2023) 22(1):157. 10.1186/s12933-023-01886-5 37386500 PMC10311747

[20] Duan SJ Ren ZY Zheng T Peng HY Niu ZH Xia H Atherogenic index of plasma combined with waist circumference and body mass index to predict metabolic-associated fatty liver disease. World J Gastroenterol. (2022) 28(36):5364–79. 10.3748/wjg.v28.i36.5364 36185625 PMC9521515

[21] Ridker PM Moorthy MV Cook NR Rifai N Lee IM Buring JE. Inflammation, cholesterol, lipoprotein(a), and 30-year cardiovascular outcomes in women. N Engl J Med. (2024) 391(22):2087–97. 10.1056/NEJMoa2405182 39216091 PMC11711015

[22] Gofton C Upendran Y Zheng MH George J. MAFLD: how is it different from NAFLD? Clin Mol Hepatol. (2022) 29(Suppl):S17. 10.3350/cmh.2022.0367 36443926 PMC10029949

[23] Virani SS Newby LK Arnold SV Bittner V Brewer LC Demeter SH 2023 AHA/ACC/ACCP/ASPC/NLA/PCNA guideline for the management of patients with chronic coronary disease: a report of the American Heart Association/American College of Cardiology Joint Committee on Clinical Practice Guidelines. Circulation. (2023) 148(9):e9–119. 10.1161/CIR.0000000000001168 37471501

[24] Vaz K Kemp W Majeed A Lubel J Magliano DJ Glenister KM NAFLD and MAFLD independently increase the risk of major adverse cardiovascular events (MACE): a 20-year longitudinal follow-up study from regional Australia. Hepatol Int. (2024) 18(4):1135–43. 10.1007/s12072-024-10706-1 39008030 PMC11297804

[25] Nayak SS Kuriyakose D Polisetty LD Patil AA Ameen D Bonu R Diagnostic and prognostic value of triglyceride glucose index: a comprehensive evaluation of meta-analysis. Cardiovasc Diabetol. (2024) 23(1):1–44. 10.1186/s12933-024-02392-y 39180024 PMC11344391

[26] Fujii H Kawada N, Japan Study Group of Nafld (JSG-NAFLD). The role of insulin resistance and diabetes in nonalcoholic fatty liver disease. Int J Mol Sci. (2020) 21(11):3863. 10.3390/ijms21113863 32485838 PMC7312931

[27] Wang A Tian X Zuo Y Chen S Meng X Wu S Change in triglyceride-glucose index predicts the risk of cardiovascular disease in the general population: a prospective cohort study. Cardiovasc Diabetol. (2021) 20(1):113. 10.1186/s12933-021-01305-7 34039351 PMC8157734

[28] Jiang W Huang G Du J Yang H Zhou S Dai D White blood cell counts can predict 4-year cardiovascular disease risk in patients with stable coronary heart disease: a prospective cohort study. Front Cardiovasc Med. (2024) 11:1358378. 10.3389/fcvm.2024.1358378 39390990 PMC11464350

[29] Duarte Lau F Giugliano RP. Lipoprotein(a) and its significance in cardiovascular disease: a review. JAMA Cardiol. (2022) 7(7):760–9. 10.1001/jamacardio.2022.0987 35583875

[30] Sasaki N Ueno Y Ozono R Nakano Y Higashi Y. Association between liver fibrosis, plasma volume status, and cardiovascular mortality: the Hiroshima Study on Glucose Metabolism and Cardiovascular Diseases. Eur J Heart Fail. (2025) 27(6):1016–24. 10.1002/ejhf.3677 40320246

[31] Song Y Dang Y Wang P Tian G Ruan L. CHD is associated with higher grades of NAFLD predicted by liver stiffness. J Clin Gastroenterol. (2020) 54(3):7. 10.1097/MCG.0000000000001238 PMC7012352 31305280

