# Supplementary material for: Development of a nomogram model for predicting coronary heart disease in patients with metabolic-associated fatty liver disease
Source: Front Cardiovasc Med. 2025 Sep 23;12:1652321. doi: 10.3389/fcvm.2025.1652321 (PMC12500640; doi:10.3389/fcvm.2025.1652321)
Supplement: Supplementary file 1 [file Table1.pdf]

Table S1. Comparison of characteristics between training and testing set, stratified by CHD status

Characteristics	Total (n = 394)	Testing set (n = 117)	Training set (n = 277)	***P***
SEX, n (%)				0.761
female	132 (34)	41 (35)	91 (33)	
male	262 (66)	76 (65)	186 (67)	
SMOKE, n (%)				0.891
0	303 (77)	91 (78)	212 (77)	
1	91 (23)	26 (22)	65 (23)	
HTN, n (%)				0.602
0	127 (32)	35 (30)	92 (33)	
1	267 (68)	82 (70)	185 (67)	
DM, n (%)				0.479
0	271 (69)	77 (66)	194 (70)	
1	123 (31)	40 (34)	83 (30)	
Age (years)	63 (56, 69)	63 (57, 68)	63 (56, 69)	0.986
BMI (kg/m^2)	26.83 (24.75, 29.05)	26.93 (24.84, 29.04)	26.83 (24.75, 29.05)	0.934
FBG (mmol/L)	6.04 (5.27, 7.36)	6.28 (5.29, 7.79)	5.89 (5.27, 7.23)	0.205
HbA1c (%)	6.30 (5.80, 7.18)	6.30 (5.70, 7.20)	6.30 (5.90, 7.10)	0.775
ALB (g/L)	41.30 ± 4.23	41.35 ± 4.00	41.28 ± 4.33	0.871
AST (U/L)	20.51 (17, 29)	20 (18, 26)	21 (17, 30)	0.499
ALT (U/L)	23 (17, 31.96)	22 (16, 31)	23 (17, 32)	0.630
TG (mmol/L)	1.54 (1.08, 2.14)	1.57 (1.07, 2.09)	1.54 (1.08, 2.15)	0.680
TC (mmol/L)	4.44 (3.59, 5.28)	4.47 (3.64, 5.27)	4.42 (3.55, 5.28)	0.721
Lp(a) (mg/L)	140.50 (61.25, 316.75)	148.00 (64, 343)	140.00 (59, 306)	0.945
HDL-C (mmol/L)	1.27 ± 0.29	1.28 ± 0.29	1.27 ± 0.29	0.793
LDL-C (mmol/L)	2.46 (1.81, 3.12)	2.54 (1.9, 3.1)	2.42 (1.79, 3.16)	0.842
FFA (mmol/L)	0.56 (0.42, 0.73)	0.57 (0.42, 0.7)	0.55 (0.42, 0.73)	0.944
BUN (mmol/L)	5.80 (4.82, 7.28)	6.05 (5.11, 7.49)	5.65 (4.73, 7.06)	0.079
Scr (umol/L)	91 (80, 103)	89 (83, 102)	91 (79, 103)	0.807
Cys-C (mg/L)	0.93 (0.82, 1.07)	0.93 (0.85, 1.06)	0.93 (0.82, 1.08)	0.694
UA (umol/L)	368.50 (305, 439.75)	368 (306, 412)	370 (304, 447)	0.257
WBC (10^9/L)	6.85 (5.87, 8.25)	6.97 (5.68, 8.83)	6.84 (5.95, 7.97)	0.564
N (10^9/L)	4.04 (3.29, 5.19)	4.04 (3.14, 5.52)	4.05 (3.35, 5)	0.68
L (10^9/L)	1.89 (1.57, 2.38)	1.88 (1.62, 2.38)	1.91 (1.55, 2.37)	0.571
PLT (10^9/L)	222 (185.25, 261.75)	229 (190, 270)	218 (181, 255)	0.100
NLR	2.07 (1.59, 2.84)	2.11 (1.57, 2.93)	2.07 (1.6, 2.79)	0.991
SII	454.17 (322.6, 678.49)	460.9 (337.84, 685.75)	453.17 (320.92, 670.5)	0.597
PNI	51.42 ± 6.02	51.89 ± 6.20	51.22 ± 5.94	0.328
TyG	1.69 (1.24, 2.24)	1.8 (1.24, 2.43)	1.68 (1.24, 2.17)	0.208
HSI	36.44 (33.05, 39.85)	36.27 (33.42, 40.43)	36.45 (33.04, 39.61)	0.779
AIP	0.06 (-0.11, 0.26)	0.05 (-0.11, 0.26)	0.06 (-0.12, 0.26)	0.876

Data are shown as means ± SD or median (quartile). Normal values were compared using paired and unpaired Student's t-tests, and non-normally distributed values were compared using Mann–Whitney tests. Statistical significance was set at ***P***<0.05. Abbreviations**:** HTN, hypertension; DM, diabetes mellitus; BMI, body mass index; FBG, Fasting plasma glucose; HbA1c, hemoglobin A1c; AST, Aspartate aminotransferase; ALT, Alanine aminotransferase; TG, Triglyceride; TC, Total cholesterol; HDL-C, High-density lipoprotein cholesterol; LDL-C, Low-density lipoprotein cholesterol; Scr serum, creatinine; Cys-C, Cystatin C; N, neutrophil; L, lymphocyte; Plt, platelet; NLR, neutrophil-to-lymphocyte ratio, SII, immune-Inflammation Index; PNI, prognostic nuteitional index; TyG, triglyceride-glucose index; HSI, hepatic steatosis index.
