# Supplementary material for: Development of a nomogram model for predicting coronary heart disease in patients with metabolic-associated fatty liver disease
Source: Front Cardiovasc Med. 2025 Sep 23;12:1652321. doi: 10.3389/fcvm.2025.1652321 (PMC12500640; doi:10.3389/fcvm.2025.1652321)
Supplement: Supplementary file 2 [file Table2.pdf]

Table S2. Comparison of Characteristics between MAFLD＋CHD Group and MAFLD Group in the training set

	Total (n = 277)	MAFLD (n = 57)	MAFLD+CHD (n = 220)	$t/\chi^2/U$	***P***
SEX, n (%)				4.595	0.032
0	91 (33)	26 (46)	65 (30)		
1	186 (67)	31 (54)	155 (70)		
SMOKE, n (%)				0.433	0.511
0	212 (77)	46 (81)	166 (75)		
1	65 (23)	11 (19)	54 (25)		
HTN, n (%)				0.204	0.651
0	92 (33)	17 (30)	75 (34)		
1	185 (67)	40 (70)	145 (66)		
DM, n (%)				0.263	0.608
0	194 (70)	42 (74)	152 (69)		
1	83 (30)	15 (26)	68 (31)		
Age, Median (Q1, Q3)	63 (56, 69)	64 (58, 69)	63 (55.75, 69)	6537.5	0.620
BMI, Mean ± SD	26.99 ± 3.08	26.75 ± 3.17	27.05 ± 3.06	-0.649	0.518
FBG, Median (Q1, Q3)	5.89 (5.27, 7.23)	5.71 (5.08, 6.51)	5.98 (5.30, 7.64)	5411.5	0.111
HbA1c, Median (Q1, Q3)	6.3 (5.90, 7.10)	6 (5.60, 6.50)	6.40 (5.90, 7.20)	4537	0.001
ALB, Mean ± SD	41.28 ± 4.33	41.91 ± 3.9	41.12 ± 4.43	1.333	0.186
AST, Median (Q1, Q3)	21 (17, 30)	20 (18, 25)	21 (17, 32)	5948.5	0.551
ALT, Median (Q1, Q3)	23 (17, 32)	22 (16, 29)	23 (17, 34)	5542	0.177
TG, Median (Q1, Q3)	1.54 (1.08, 2.15)	1.5 (1.07, 2.01)	1.54 (1.10, 2.20)	5988.5	0.602
TC, Median (Q1, Q3)	4.42 (3.55, 5.28)	4.29 (3.51, 5.08)	4.45 (3.58, 5.30)	5698	0.289
Lp(a), Median (Q1, Q3)	140 (59, 306)	119 (57, 246)	145.50 (60.50, 346.75)	5363.5	0.093
HDL-C, Mean ± SD	1.27 ± 0.29	1.31 ± 0.25	1.26 ± 0.30	1.141	0.257
LDL-C, Median (Q1, Q3)	2.42 (1.79, 3.16)	2.13 (1.75, 2.95)	2.50 (1.79, 3.19)	5362	0.092
FFA, Median (Q1, Q3)	0.55 (0.42, 0.73)	0.59 (0.45, 0.71)	0.53 (0.39, 0.74)	6776	0.348
BUN, Median (Q1, Q3)	5.65 (4.73, 7.06)	5.37 (4.66, 6.51)	5.74 (4.76, 7.25)	5734.5	0.321
Scr, Median (Q1, Q3)	91 (79, 103)	82 (78, 101)	93 (81.75, 103)	5085	0.028
Cys-C, Median (Q1, Q3)	0.93 (0.82, 1.08)	0.85 (0.77, 0.98)	0.95 (0.83, 1.12)	4572.5	0.002
UA, Median (Q1, Q3)	370 (304, 447)	339 (303, 415)	386 (312.75, 449.25)	5353	0.089
WBC, Median (Q1, Q3)	6.84 (5.95, 7.97)	6.31 (5.47, 7.32)	6.99 (6.04, 8.19)	4710	0.004
N, Median (Q1, Q3)	4.05 (3.35, 5.00)	3.64 (3.26, 4.64)	4.14 (3.38, 5.09)	5175.5	0.042
L, Median (Q1, Q3)	1.91 (1.55, 2.37)	1.78 (1.50, 2.37)	1.93 (1.56, 2.38)	5657.5	0.256
PLT, Median (Q1, Q3)	218 (181, 255)	215 (197, 262)	218 (180.5, 253.50)	6540	0.617
NLR, Median (Q1, Q3)	2.07 (1.60, 2.79)	2.03 (1.67, 2.51)	2.07 (1.59, 2.88)	5951	0.555
SII, Median (Q1, Q3)	453.17 (320.92,670.5)	452.36 (331, 626.36)	453.45 (318.70, 682.37)	6091.5	0.741
PNI, Mean ± SD	51.22 ± 5.94	51.30 ± 5.09	51.2 ± 6.16	0.126	0.900
TyG, Median (Q1, Q3)	1.68 (1.24, 2.17)	1.18 (0.71, 1.71)	1.75 (1.35, 2.35)	3265.5	< 0.001
HSI, Mean ± SD	36.63 ± 5.25	36.62 ± 4.91	36.63 ± 5.34	-0.017	0.987
AIP, Median (Q1, Q3)	0.06 (-0.12, 0.26)	-0.14 (-0.49, -0.01)	0.1 (-0.05, 0.31)	2643.5	< 0.001
