# Supplementary material for: Development of a nomogram model for predicting coronary heart disease in patients with metabolic-associated fatty liver disease
Source: Front Cardiovasc Med. 2025 Sep 23;12:1652321. doi: 10.3389/fcvm.2025.1652321 (PMC12500640; doi:10.3389/fcvm.2025.1652321)
Supplement: Supplementary file 3 [file Table3.docx]

**Table S3. Binary logistic regression analysis of MAFLD Complicated with CHD in the training set**

|  | **Multivariate** | | | | **Multivariate** | | | |
| --- | --- | --- | --- | --- | --- | --- | --- | --- |
| **Variables** | **B** | **SE** | **OR (95% CI)** | ***P*** | **B** | **SE** | **OR (95% CI)** | ***P*** |
| Sex (male) | 0.808 | 0.377 | 2.244 (1.074~4.749) | 0.032 | 0.737 | 0.371 | 2.089 (1.011~4.355) | 0.047 |
| Lp (a) | 0.002 | 0.001 | 1.002 (1.001~1.005) | 0.012 | 0.003 | 0.001 | 1.003 (1.001~1.005) | 0.008 |
| LDL-C | 0.313 | 0.196 | 1.367 (0.945~2.041) | 0.110 |  |  |  |  |
| WBC | 0.257 | 0.120 | 1.294 (1.036~1.657) | 0.031 | 0.274 | 0.120 | 1.315 (1.053~1.684) | 0.022 |
| TyG | 0.629 | 0.308 | 1.875 (1.052~3.53) | 0.041 | 0.646 | 0.310 | 1.908 (1.066~3.614) | 0.037 |
| AIP | 4.020 | 0.842 | 55.727 (11.443~315.764) | <0.001 | 4.050 | 0.850 | 57.376 (11.578~330.134) | <0.001 |

Values are expressed as mean (95% confidence interval). **Abbreviations:** B:​​ Unstandardized regression coefficient. SE:​​ Standard error of the unstandardized coefficient. OR:​​ Odds ratio. An OR > 1 indicates increased odds of the outcome, while an OR < 1 indicates decreased odds, per one-unit increase in the predictor variable.
